# Airway management and anaesthetic interventions for children with cleft lip and/or palate undergoing general anaesthesia: Protocol for a systematic review and meta-analysis

**DOI:** 10.1371/journal.pone.0361327

**Published:** 2026-10-27

**Authors:** Sharmin Akter Sumi, Kazi Mahzabin Arin, Shahriar Mohd Shams, Tanzila Rafique, Keertika Orchi, Arna Chowdhury, Mohammad Meshbahur Rahman, Md Atiqul Haque

**Affiliations:** 1 Department of Anatomy, Bangladesh Medical University, Dhaka, Bangladesh; 2 Department of Anaesthesia, Analgesia and Intensive Care Medicine, Bangladesh Medical University, Dhaka, Bangladesh; 3 Department of Orthodontics, Bangladesh Medical University, Dhaka, Bangladesh; 4 Department of Public Health and Informatics, Bangladesh Medical University, Dhaka, Bangladesh; 5 Department of Biostatistics, National Institute of Preventive and Social Medicine, Dhaka, Bangladesh; Sapienza University of Rome: Universita degli Studi di Roma La Sapienza, ITALY

## Abstract

**Background:**

Children with cleft lip and/or palate may present unique challenges during perioperative airway management because of altered upper-airway and craniofacial anatomy. These children frequently undergo surgical procedures requiring general anaesthesia and airway instrumentation. Several airway-management and anaesthetic techniques have been evaluated to facilitate tracheal intubation and reduce perioperative airway complications, but the comparative evidence from randomised controlled trials has not been systematically evaluated.

**Methods:**

This systematic review and meta-analysis will include randomised controlled trials involving children < 18 years with cleft lip and/or palate undergoing general anaesthesia. Eligible studies will compare alternative techniques or devices for airway management or other anaesthetic strategies between two groups of cleft lip/palate patients. The primary outcome will be difficulty in tracheal intubation. Where definitions vary substantially, outcomes will be analysed separately or synthesised narratively. Secondary outcomes will include difficult mask ventilation, oxygen desaturation, airway obstruction, difficult laryngoscopy, first-attempt intubation success, number of intubation attempts, time to successful intubation or airway establishment, failed intubation and other perioperative airway or respiratory adverse events. Two reviewers will independently select RCTs, extract data and assess risk of bias. Risk of bias will be evaluated using the Cochrane Risk of Bias 2 tool. Random-effects meta-analysis will be performed where clinically appropriate. Risk ratios will be used for dichotomous outcomes and mean differences or standardized mean differences for continuous outcomes. Certainty of evidence for important outcomes will be assessed using the Grading of Recommendations Assessment, Development and Evaluation (GRADE) approach.

**Registration and review status:**

The review was prospectively registered in PROSPERO on 10 March 2026 (CRD420261326076). Full-text screening and data extraction are ongoing and not yet complete. Protocol amendments are documented transparently in PROSPERO.

## Introduction

Cleft lip and/or palate are among the most common congenital craniofacial anomalies worldwide and result from disruption of normal fusion of facial structures during embryonic development [1,2]. Affected children display substantial variation in maxillary, palatal, nasal and midfacial anatomy. These anatomical characteristics may influence upper-airway configuration and have important implications when general anaesthesia and airway instrumentation are required.

Children with cleft lip and/or palate commonly undergo several surgical procedures during infancy and childhood, including cleft lip repair, palatoplasty, alveolar bone grafting and secondary reconstructive procedures. Consequently, some children experience repeated exposure to general anaesthesia and airway management. Airway management in young children is already physiologically demanding because of smaller airway dimensions, reduced oxygen reserves and rapid desaturation. Altered oral and craniofacial anatomy associated with cleft conditions may further complicate mask ventilation, laryngoscopy, tracheal intubation and maintenance of a secure airway [3–5].

Different approaches have therefore been investigated to improve airway management during cleft surgery. Randomised trials have compared, among other strategies, hard-gum-shield-assisted versus paraglossal intubation [6], Airway Scope versus Macintosh laryngoscopy [7], videolaryngoscopic versus direct paraglossal laryngoscopy [8], and different preformed endotracheal tube designs [9]. Outcomes evaluated across these trials include intubation time, first-attempt success, laryngoscopic view, intubation difficulty, need for external laryngeal manipulation, tube exchange, oxygen desaturation and airway-related complications. However, variation in interventions and outcome definitions makes individual trial findings difficult to translate into an overall evidence-based approach.

Recent systematic reviews have synthesized observational evidence concerning pharyngeal airway dimensions in individuals with cleft lip and palate [10,11]. These reviews improve understanding of structural airway differences but address a question distinct from the comparative effectiveness and safety of airway-management and anaesthetic interventions. For example, a recent meta-analysis of 39 studies focused primarily on pharyngeal airway volumes and cross-sectional dimensions rather than randomised airway-management strategies [10]. Therefore, a systematic evaluation focused specifically on randomised intervention evidence remains clinically relevant. Relevant craniofacial or airway anatomical features reported in eligible trials will be presented as participant baseline characteristics or potential effect modifiers rather than independent primary outcomes.

The primary objective of this systematic review and meta-analysis is to assess the comparative effectiveness of airway management and anaesthetic interventions for tracheal intubation in paediatric patients with cleft lip and/or palate undergoing general anaesthesia. Secondary objectives are to evaluate first-attempt intubation success, difficult laryngoscopy, intubation time, difficult mask ventilation and other perioperative airway or respiratory adverse event outcomes.

## Methods

### Study design and reporting framework

This is a protocol for systematic review and meta-analysis of randomised controlled trials. The protocol will be reported in accordance with the Preferred Reporting Items for Systematic Reviews and Meta-Analyses Protocols (PRISMA-P) 2015 statement [12]. The completed systematic review and meta-analysis will be reported according to the PRISMA 2020 statement [13]. The review is prospectively registered with the International Prospective Register of Systematic Reviews (PROSPERO; CRD420261326076) on 10 March 2026. The registered review start date was 1 May 2026. All studies meeting the predetermined eligibility criteria will be considered for inclusion.

### Protocol amendments

Following registration in PROSPERO and during methodological refinement of the review, the eligibility criteria were clarified to include only randomised controlled trials. Grading of Recommendations Assessment, Development and Evaluation (GRADE) was added to assess the certainty of evidence. These amendments were made before quantitative evidence synthesis and were not based on the magnitude, direction or statistical significance of pooled treatment effects. All substantive amendments will be dated, justified and updated in the PROSPERO record and will be reported transparently in the completed systematic review.

### Review question

The review will address the following question:

In children aged up to 18 years with cleft lip and/or palate undergoing general anaesthesia, what is the comparative effectiveness and safety of different airway-management or anaesthetic interventions?

### Eligibility criteria

#### Population.

Eligible studies will include children aged up to 18 years diagnosed with cleft lip, cleft palate, or cleft lip-palate who undergo a surgical or procedural intervention requiring general anaesthesia and airway management.

In accordance with the registered protocol, studies focusing primarily on preterm infants or on major syndrome or developmental craniofacial disorders other than cleft lip and/or palate will be excluded unless relevant data for the eligible cleft population can be extracted separately.

#### Interventions.

Primary domain: airway-management interventions

Secondary: associated anaesthetic strategies affecting airway outcomes.

#### Comparators.

Eligible comparators will include usual or conventional airway management, another airway-management technique or device, or an alternative anaesthetic strategy evaluated within the randomised trial.

#### Study design.

Only randomised controlled trials will be included.

#### Publication characteristics.

Peer-reviewed full-text studies published in English will be eligible. The registered search period begins on 1 January 2001. The final search will therefore cover 1 January 2001 to the date of the final search update.

### Outcomes

#### Primary outcome.

The primary outcome will be difficult tracheal intubation, as defined by the investigators in each eligible trial. If difficult intubation cannot be pooled due to inconsistent definitions, first-attempt success and number of attempts will be considered supportive outcomes. Where available, the definition or grading system used to classify intubation difficulty will be recorded, including number of attempts, Intubation Difficulty Score, investigator-defined difficulty, or failed tracheal intubation.

#### Secondary outcomes.

Secondary outcomes will include first-attempt or first-pass intubation success; failed tracheal intubation; difficult laryngoscopy; Cormack-Lehane or comparable laryngoscopic grade; number of intubation attempts; time to successful tracheal intubation or airway establishment; requirement for external laryngeal manipulation or additional airway manoeuvres; difficult or failed mask ventilation; tracheal-tube exchange or displacement where applicable; oxygen desaturation or hypoxaemia; airway obstruction; laryngospasm; bronchospasm; aspiration; airway, oral or oropharyngeal trauma; postoperative airway morbidity; and other clearly defined perioperative airway or respiratory adverse events.

Relevant baseline anatomical features, including cleft phenotype, micrognathia or other reported airway-related craniofacial characteristics, will be extracted as participant characteristics and considered as potential sources of clinical heterogeneity where sufficient information is available.

### Information sources

Electronic database searches were conducted in the Cochrane Library, Embase, PubMed, Scopus, Web of Science and Google Scholar for studies published from 1st January 2001–31st May 2026. To identify relevant registered, ongoing or completed randomised controlled trials, ClinicalTrials.gov and the World Health Organization (WHO) International Clinical Trials Registry Platform were searched. Backward and forward citation searching was also performed on the included studies.

### Search strategy

The search strategy combined controlled vocabulary and free-text terms representing four concepts: cleft lip/palate, airway management or anaesthesia, children, and randomised controlled trials. Table 1 presents the detailed search terms and Medical Subject Heading (MeSH) strategies used across the databases.

**Table 1:** Search term strategies using keywords and Medical Subject Heading (MeSH) terms.

Keywords	MeSH Terms
anesthetic implications	(Anaesthetic implication) OR (anaesthetic complication) OR (anaesthetic management) OR (Preoperative Care) OR (Postoperative Complications)
airway	(Airway) OR (upper airway) OR (airway instrumentation) OR (airway obstruction) OR (airway instrumentation) OR (airway adverse effect) OR (airway adverse events) OR (airway management)
craniofacial anatomy	(Craniofacial) OR (Anatomical abnormalities) OR (craniofacial abnormalities) OR (craniofacial anomalies) OR (Maxillofacial Abnormalities)
cleft lip & cleft palate	(Cleft lip palate) OR (Cleft lip-palate) OR (cleft lip/palate) OR (cleft lip) OR (cleft palate) OR (orofacial cleft)
children	(newborn) OR (infant) OR (preschool Child) OR (paediatrics) OR (adolescent)

The strategy was adapted to the syntax and indexing requirements of each database. Search strategies, final search dates and numbers of records retrieved from each database will be reported in the completed review.

### Data management

Search results will be imported into Rayyan for record management, deduplication and screening. Microsoft Excel will be used for structured data extraction. Separate files or worksheets will be maintained for study identification, eligibility decisions, reasons for full-text exclusion, extracted study characteristics, outcome data and risk-of-bias judgments. Version-controlled copies of the extraction file will be retained to preserve an audit trail of changes made during the review.

### Study selection

Two reviewers will screen titles and abstracts independently following the prespecified eligibility criteria. Studies considered potentially eligible, or for which eligibility cannot be confirmed from the title and abstract, will undergo full-text assessment by two reviewers independently. Full-text eligibility decisions will be based exclusively on the prespecified population, intervention, comparator and study-design criteria. The reasons for exclusion will be recorded at the full-text stage. Disagreements will be resolved through discussion and consensus. Where consensus cannot be reached, a third reviewer will adjudicate. The completed review will report on the study-selection process using a PRISMA flow diagram.

### Data extraction

A standardized Microsoft Excel data-extraction form will be used and pilot tested before completion of extraction. Data extraction will be performed independently and in duplicate. Extraction responsibilities may be distributed among four reviewers, but each eligible study will be evaluated by two reviewers independently. Disagreements will be resolved through discussion or consultation with a third reviewer.

The following information will be extracted where available: publication details; country and clinical setting; study design; recruitment period; sample size; participant age, sex and cleft phenotype; relevant baseline craniofacial or airway characteristics; surgical procedure; intervention characteristics; comparator characteristics; anaesthetic induction and maintenance techniques; airway device or intubation method; definitions and measurement methods for outcomes; duration of follow-up where relevant; numerical outcome data; adverse events; funding or sponsorship of the individual trial where reported; and information needed for risk-of-bias assessment.

### Missing or unclear information

The review will primarily use information available from published articles, supplementary files and trial-registry records. In accordance with the registered protocol, study authors will not routinely be contacted to obtain additional information. Missing or unclear information will be documented and considered during risk-of-bias assessment and interpretation of the evidence.

Where statistical information such as standard deviations is not reported but can be derived from other available statistics using established methods, appropriate calculations described in the Cochrane Handbook will be considered [14].

### Risk of bias assessment

Two reviewers will assess risk of bias independently using the Cochrane Risk of Bias 2 tool. The assessment will consider bias arising from the randomisation technique, any deviation from the intended interventions, missing data and selection of the reported result. Each relevant result will be categorized as having low risk of bias, some concerns, or high risk of bias in accordance with RoB 2 guidance. Disagreements will be resolved through discussion or adjudication by a third reviewer. Because only randomised controlled trials will be included in the final review, ROBINS-I and JBI critical-appraisal tools will not be used.

### Data synthesis

A structured narrative synthesis will be presented for all eligible studies. Meta-analysis will be undertaken only when at least two studies are sufficiently comparable with respect to participants, interventions, comparators and outcome definitions. Studies evaluating clinically distinct intervention classes will not be pooled merely because they report the same outcome.

For dichotomous outcomes, including difficult intubation, first-attempt success, failed intubation and adverse events, treatment effects will generally be expressed as risk ratios with 95% confidence intervals. For continuous outcomes, such as intubation time, mean differences with 95% confidence intervals will be calculated when the same measurement scale is used. Standardized mean differences will be considered when studies measure the same underlying construct using different scales.

Random-effects meta-analysis will be used as the primary synthesis model where quantitative pooling is appropriate because variation in patient age, cleft phenotype, surgical procedures, airway techniques and anaesthetic interventions is anticipated. Between-study variance will preferably be estimated using restricted maximum likelihood methods. Where quantitative synthesis is inappropriate because of insufficient studies or substantial clinical or methodological diversity, findings will be summarized narratively and presented in structured tables. Statistical heterogeneity will be assessed using the I² statistic and τ² estimate, and clinical and methodological heterogeneity will be considered when interpreting pooled effects. Analyses will be conducted using established meta-analysis software such as Review Manager and/or R. The exact software and version will be reported in the completed review.

Because eligible trials are expected to compare heterogeneous airway techniques and devices (for example, videolaryngoscopy versus direct laryngoscopy, or different laryngoscope designs), trials will be pooled only when interventions, comparators and outcome definitions are sufficiently similar. Trials without a shared comparator will not be combined, and network meta-analysis will not be undertaken because the evidence network is expected to be sparse. Time to intubation will be interpreted together with success-related outcomes (first-attempt success, number of attempts and failed intubation) and airway complications, which will be tabulated side by side for each comparison; a shorter intubation time will not be regarded as beneficial if it is accompanied by lower success or more complications.

### Unit-of-analysis considerations

For conventional parallel-group randomised trials, the individual participant will be the unit of analysis. For trials containing more than two relevant intervention groups, eligible arms will be included while avoiding double counting of shared comparator participants. Where necessary, comparator groups may be divided, or intervention groups may be combined according to established Cochrane methods [14].

Trials with zero events in both intervention groups will be described but will not contribute directly to conventional relative effect estimates where these cannot be calculated.

### Subgroup analyses

Where sufficient numbers of clinically comparable trials are available, exploratory subgroup analyses may be undertaken according to age group, cleft phenotype, type of cleft surgery, and category of airway-management or anaesthetic intervention. Subgroup analyses will only be undertaken where adequate data are available and will be interpreted cautiously because such analyses may have limited statistical power. Meta-regression is not prespecified because the number of eligible randomised trials is expected to be limited.

### Sensitivity analyses

When sufficient studies are accessible, we will conduct sensitivity analyses to evaluate the robustness of the findings. These may include exclusion of trials assessed as high risk of bias and assessing the effect of alternative reasonable analytical assumptions. Sensitivity analyses must be interpreted with caution, particularly when the meta-analysis is based on a limited number of trials.

### Assessment of reporting bias

If at least 10 sufficiently comparable randomised trials contribute to a meta-analysis, funnel plots will be examined for potential small-study effects. Where appropriate, we will consider a statistical test of funnel-plot asymmetry, such as Egger’s test. We will not interpret funnel-plot asymmetry as publication bias without considering other clinical and methodological explanations.

### Certainty of evidence

We will assess the certainty of evidence for the primary and secondary outcomes using the Grading of Recommendations Assessment, Development and Evaluation approach (GRADE) [15].

Randomised trial evidence will initially be considered high certainty and may be rated down because of risk of bias, inconsistency or publication bias. Certainty will be categorized as high, moderate, low or very low. A Summary of Findings table will be prepared for the comparisons where appropriate.

### Review status and timeline

The systematic review was registered prospectively in PROSPERO on 10 March 2026 (CRD420261326076), before the registered review start date of 1 May 2026. At the time of preparation of this revised protocol, full-text screening and data extraction are ongoing and have not been completed.

No quantitative evidence synthesis or meta-analysis has yet been performed, and no pooled review results have been generated. The registered review timeline specifies 31 August 2026 as the anticipated review end date. Data extraction is currently expected to be completed by approximately this date, with evidence synthesis and preparation of the completed systematic review to follow. Any change to the anticipated completion timeline will be updated in PROSPERO.

### Ethics and dissemination

This review will collect data from published studies and will not involve direct recruitment of human participants, collection of identifiable personal information, or assignment to a new intervention. Therefore, this systematic review does not require institutional ethics approval or individual informed consent. The findings will be submitted to a peer-reviewed journal for publication and disseminated through scientific seminars and academic presentations.

## Discussion

Children with cleft lip and/or palate increase the complexity of perioperative airway and anaesthetic management due to the anatomical variation and repeated surgical procedures. Although several randomised trials have evaluated different airway-management devices, intubation approaches, endotracheal tubes and anaesthetic strategies, the clinical literature remains fragmented across different interventions and outcome measures.

The proposed systematic review will focus specifically on randomised controlled trial evidence, allowing a more direct assessment of comparative intervention effects than would be possible from observational studies alone. Restricting the review to randomised trials is also expected to provide a more methodologically coherent evidence base for evaluating intervention effectiveness and safety.

Airway management in children with cleft lip and/or palate is clinically important because successful, smooth airway establishment is essential for safe surgery and preventing perioperative airway adverse events. The anatomical variations associated with cleft lip and/or palate, including altered oral cavity structures, palatal defects and potential differences in airway anatomy, may influence the effectiveness and safety of commonly used airway management techniques. Although several devices and approaches have been introduced to improve intubation success rate and reduce airway-related complications, anaesthesiologists currently rely on evidence from individual trials with varying interventions. A systematic synthesis of randomised evidence is therefore needed to clarify the comparative benefits and potential limitations of available airway-management and anaesthetic strategies, supporting more evidence-informed decision-making in airway management in children with cleft lip and/or palate.

The review nevertheless has anticipated limitations. The number of eligible randomised trials may be small, interventions may be clinically diverse, and definitions of difficult intubation, intubation success and airway complications may vary among studies. These factors may limit the number of outcomes for which quantitative synthesis is appropriate. Language restriction to English and the registered publication-date restriction beginning in 2001 may also introduce selection limitations. These limitations will be considered when interpreting the findings and when evaluating certainty of evidence.

The review is expected to identify which airway-management and anaesthetic interventions have been tested in randomised trials, and clarify their comparative effectiveness on airway anaesthetic management outcomes.

## Supporting information

S1 Checklist PRISMA-P 2015 checklist. (DOCX)
