# Supplementary material for: Airway management and anaesthetic interventions for children with cleft lip and/or palate undergoing general anaesthesia: Protocol for a systematic review and meta-analysis
Source: PLoS One. 2026 Oct 27;21(10):e0361327. doi: 10.1371/journal.pone.0361327 (PMC13712514; doi:10.1371/journal.pone.0361327)
Supplement: Supplementary file 1 [file pone.0361327.s001.docx]

**S1 Checklist. PRISMA-P 2015 (Preferred Reporting Items for Systematic Review and Meta-Analysis Protocols) Checklist**

| **Section & Topic** | **Item No.** | **Checklist Item** | **Reported on Page #, Line #** |
| --- | --- | --- | --- |
| **ADMINISTRATIVE INFO** |  |  |  |
| Title — Identification | 1a | Identify the report as a protocol of a systematic review | Page 1, Line 3 |
| Title — Update | 1b | If the protocol is for an update of a previous systematic review, identify as such | Not applicable (not an update protocol) |
| Registration | 2 | If registered, provide the name of the registry (such as PROSPERO) and registration number | Page 3, Lines 48-50; Page 5, Lines 100–102 |
| Authors — Contact | 3a | Provide name, institutional affiliation, e-mail address of all protocol authors; provide physical mailing address of corresponding author | Page 1, Lines 6–22 |
| Authors — Contributions | 3b | Describe contributions of protocol authors and identify the guarantor of the review | Reported in the PLOS ONE submission system |
| Amendments | 4 | If the protocol represents an amendment of a previously completed or published protocol, identify as such and list changes; otherwise, state plan for documenting important protocol amendments | Page 5, Lines 104–110 |
| Support — Sources | 5a | Indicate sources of financial or other support for the review | Reported in the PLOS ONE submission system |
| Support — Sponsor | 5b | Provide name for the review funder and/or sponsor | Reported in the PLOS ONE submission system |
| Support — Role of Sponsor or Funder | 5c | Describe roles of funder(s), sponsor(s), and/or institution(s), if any, in developing the protocol | Reported in the PLOS ONE submission system |
| **INTRODUCTION** |  |  |  |
| Rationale | 6 | Describe the rationale for the review in the context of what is already known | Pages 3–4, Lines 62–78 |
| Objectives | 7 | Provide an explicit statement of the question(s) the review will address with reference to participants, interventions, comparators, and outcomes (PICO) | Page 6, Lines 117–130; Page 7, Lines 138–156 |
| **METHODS** |  |  |  |
| Eligibility Criteria | 8 | Specify the study characteristics (such as PICO, study design, setting, time frame) and report characteristics (such as years considered, language, publication status) to be used as criteria for eligibility for the review | Pages 6–8, Lines 116–163 |
| Information Sources | 9 | Describe all intended information sources (such as electronic databases, contact with study authors, trial registers or other grey literature sources) with planned dates of coverage | Page 8, Lines 157–163 |
| Search Strategy | 10 | Present draft of search strategy to be used for at least one electronic database, including planned limits, such that it could be repeated | Pages 8–9, Lines 164–173 |
| Study Records — Data management | 11a | Describe the mechanism(s) that will be used to manage records and data throughout the review | Page 9, Lines 174–179 |
| Study Records — Selection process | 11b | State the process that will be used for selecting studies (such as two independent reviewers) through each phase of the review (that is, screening, eligibility and inclusion in meta-analysis) | Page 9, Lines 180–188 |
| Study Records — Data collection process | 11c | Describe planned method of extracting data from reports (such as piloting forms, done independently, in duplicate), any processes for obtaining and confirming data from investigators | Page 10-11, Lines 203–218 |
| Data Items | 12 | List and define all variables for which data will be sought (such as PICO items, funding sources), any pre-planned data assumptions and simplifications | Page 11-12, Lines 219–239 |
| Outcomes and Prioritization | 13 | List and define all outcomes for which data will be sought, including prioritization of main and additional outcomes, with rationale | Pages 7–8, Lines 137-156; |
| Risk of Bias | 14 | Describe anticipated methods for assessing risk of bias of individual studies, including whether this will be done at the outcome or study level, or both; state how this information will be used in data synthesis | Page 11-12, Lines 211–239 |
| Data Synthesis | 15a | Describe criteria under which study data will be quantitatively synthesised | Page 11, Lines 219–239 |
|  | 15b | If data are appropriate for quantitative synthesis, describe planned summary measures, methods of handling data and methods of combining data from studies, including any planned exploration of consistency (such as I², Kendall's τ) | Pages 11–12, Lines 230–239 |
|  | 15c | Describe any proposed additional analyses (such as sensitivity or subgroup analyses, meta-regression) | Page 12-13, Lines 248–269 |
|  | 15d | If quantitative synthesis is not appropriate, describe the type of summary planned | Page 11-12, Lines 230–239 |
| Meta-bias(es) | 16 | Specify any planned assessment of meta-bias(es) (such as publication bias across studies, selective reporting within studies) | Page 13, Lines 260-264 |
| Confidence in Evidence | 17 | Describe how the strength of the body of evidence will be assessed (such as GRADE) | Page 13, Lines 265–271 |

**It is strongly recommended that this checklist be read in conjunction with the PRISMA-P Explanation and Elaboration for important clarification on the items. Amendments to a review protocol should be tracked and dated. The copyright for PRISMA-P (including checklist) is held by the PRISMA-P Group and is distributed under a Creative Commons Attribution Licence 4.0.*

*From: Shamseer L, Moher D, Clarke M, Ghersi D, Liberati A, Petticrew M, Shekelle P, Stewart L, PRISMA-P Group. Preferred reporting items for systematic review and meta-analysis protocols (PRISMA-P) 2015: elaboration and explanation. BMJ. 2015 Jan 2;349(jan02 1):g7647.*
