# Supplementary material for: Airway management and anaesthetic interventions for children with cleft lip and/or palate undergoing general anaesthesia: Protocol for a systematic review and meta-analysis
Source: PLoS One. 2026 Oct 27;21(10):e0361327. doi: 10.1371/journal.pone.0361327 (PMC13712514; doi:10.1371/journal.pone.0361327)
Supplement: Supplementary file 2 [file pone.0361327.s002.docx]

**Response to Reviewers**

**Manuscript ID:** PONE-D-26-43191
**Title:** Airway management and anaesthetic interventions for children with cleft lip and/or palate undergoing general anaesthesia: protocol for a systematic review and meta-analysis

Dear Dr. Silvia Fiorelli and Reviewers,

Thank you for the careful evaluation of our manuscript and for the constructive comments. We have revised the protocol in response to all points raised. Below is our point-by-point response. All changes are highlighted in the “Revised Manuscript with Track Changes” file. A clean version of the revised manuscript is also provided.

**Journal Requirements**

**1. PLOS ONE style requirements**
We have carefully reviewed the PLOS ONE formatting templates and revised the manuscript (title page, headings, spacing, and overall formatting) to ensure full compliance with the journal’s style requirements. In addition, the name of the reporting guideline for protocols has been corrected to “(PRISMA-P) 2015” (Methods: Study design and reporting framework).

**2. Data Availability Statement**
We have clarified our data-sharing plan. Upon completion of the systematic review, the study-level extraction dataset, risk-of-bias assessments, and data underlying any meta-analytic estimates will be made available as Supporting Information. The Data Availability Statement in the manuscript has been updated accordingly.

**3. Reference to Table 1**
We thank the editors for pointing this out. We have now explicitly referred to Table 1 in the main text (Search strategy section). The sentence now reads:
The detailed search terms and Medical Subject Heading (MeSH) strategies used are presented in Table 1.

**4. Recommended citations**
None of the reviewers recommended citation of specific additional works. No changes were required.

**5. Reference list**
We have carefully reviewed the reference list for completeness, accuracy, and currency. No retracted articles were identified. No changes to the reference list were required.

**Reviewer #1**

We thank Reviewer #1 for the positive assessment of the protocol.

**Reviewer #2**

We sincerely thank Reviewer #2 for the thoughtful and constructive comments regarding the methodological challenges of synthesising heterogeneous airway trials.

**Comment:**
The reviewer noted that many trials compare different airway techniques without a consistent baseline control, that primary and secondary outcomes often differ across studies, and that outcomes such as time to intubation and success rate can be complex to interpret together. The reviewer suggested discussion of how these issues will be handled.

**Response:**
We fully agree with these important points. In response, we have substantially strengthened the **Data synthesis** section to make our planned approach explicit:

- Studies will be grouped for quantitative synthesis only when interventions and comparators are judged sufficiently similar.
- Trials comparing different classes of devices without a shared control arm will not be pooled in a single pairwise meta-analysis.
- Where data allow, exploratory subgroup analyses by intervention category (e.g., videolaryngoscopy versus direct laryngoscopy, specific device designs, or anaesthetic strategies) will be considered.
- When quantitative synthesis is inappropriate because of clinical diversity, results will be presented narratively and in structured tables organised by intervention type and outcome.
- Continuous outcomes such as time to intubation will be interpreted jointly with dichotomous success-related outcomes (first-attempt success, number of attempts, and failed intubation). A shorter mean intubation time will not be interpreted in isolation if accompanied by lower success rates or higher complication rates.
- Network meta-analysis will not be undertaken because the evidence network is expected to be sparse, and trials without a shared comparator will not be combined.
- Time to intubation, success-related outcomes and airway complications will be tabulated side by side for each comparison, so that speed and success can be judged together.
- A corresponding sentence has been added to the limitations paragraph of the Discussion.

These additions directly address the reviewer’s concerns about heterogeneity of techniques, lack of a common baseline, and the clinical interpretation of time versus success outcomes. We believe the revised synthesis plan is now more transparent and methodologically robust.

**Reviewer #3**

We thank Reviewer #3 for the positive evaluation of the research question and methods.

We hope that the revisions satisfactorily address all comments. We remain grateful for the reviewers’ and editor’s time and expertise, which have improved the clarity and methodological rigour of this protocol.

Kind regards,

**Kazi Mahzabin Arin**
(on behalf of all co-authors)
Corresponding author
Department of Anaesthesia, Analgesia and Intensive Care Medicine
Bangladesh Medical University, Shahbag, Dhaka, Bangladesh
Email: [kazimahzabinarin@bsmmu.edu.bd](mailto:kazimahzabinarin@bsmmu.edu.bd)
