# Supplementary material for: Airway management and anaesthetic interventions for children with cleft lip and/or palate undergoing general anaesthesia: Protocol for a systematic review and meta-analysis
Source: PLoS One. 2026 Oct 27;21(10):e0361327. doi: 10.1371/journal.pone.0361327 (PMC13712514; doi:10.1371/journal.pone.0361327)
Supplement: Peer review history

# Peer review: Decision Letter 0

**DOI:** 10.1371/journal.pone.0361327.r001

**Authors:** Silvia Fiorelli

16 Sep 2026

Dear Dr. Arin,

Thank you for submitting your manuscript to PLOS One. After careful consideration, we feel that it has merit but does not fully meet PLOS One’s publication criteria as it currently stands. Therefore, we invite you to submit a revised version of the manuscript that addresses the points raised during the review process.

==============================

**ACADEMIC EDITOR:**

==============================

Please submit your revised manuscript by Oct 01 2026 11:59PM. If you will need more time than this to complete your revisions, please reply to this message or contact the journal office at plosone@plos.org. When you're ready to submit your revision, log on to https://www.editorialmanager.com/pone/ and select the 'Submissions Needing Revision' folder to locate your manuscript file.

- A letter that responds to each point raised by the academic editor and reviewer(s). You should upload this letter as a separate file labeled 'Response to Reviewers'.
- A marked-up copy of your manuscript that highlights changes made to the original version. You should upload this as a separate file labeled 'Revised Manuscript with Track Changes'.
- An unmarked version of your revised paper without tracked changes. You should upload this as a separate file labeled 'Manuscript'.

If you would like to make changes to your financial disclosure, please include your updated statement in your cover letter. Guidelines for resubmitting your figure files are available below the reviewer comments at the end of this letter.

If applicable, we recommend that you deposit your laboratory protocols in protocols.io to enhance the reproducibility of your results. Protocols.io assigns your protocol its own identifier (DOI) so that it can be cited independently in the future. For instructions see: https://journals.plos.org/plosone/s/submission-guidelines#loc-laboratory-protocols. Additionally, PLOS One offers an option for publishing peer-reviewed Lab Protocol articles, which describe protocols hosted on protocols.io. Read more information on sharing protocols at https://plos.org/protocols?utm_medium=editorial-email&utm_source=authorletters&utm_campaign=protocols.

As the corresponding author, your ORCID iD is verified in the submission system and will appear in the published article. PLOS supports the use of ORCID, and we encourage all coauthors to register for an ORCID iD and use it as well. Please encourage your coauthors to verify their ORCID iD within the submission system before final acceptance, as unverified ORCID iDs will not appear in the published article. *Only*  the individual author can complete the verification step; PLOS staff *cannot*  verify ORCID iDs on behalf of authors.

We look forward to receiving your revised manuscript.

Kind regards,

Silvia Fiorelli

Academic Editor

PLOS One

Journal Requirements:

When submitting your revision, we need you to address these additional requirements.

1. Please ensure that your manuscript meets PLOS ONE's style requirements, including those for file naming. The PLOS ONE style templates can be found at

https://journals.plos.org/plosone/s/file?id=wjVg/PLOSOne_formatting_sample_main_body.pdf and

https://journals.plos.org/plosone/s/file?id=ba62/PLOSOne_formatting_sample_title_authors_affiliations.pdf

2. When completing the data availability statement of the submission form, you indicated that you will make your data available on acceptance. We strongly recommend all authors decide on a data sharing plan before acceptance, as the process can be lengthy and hold up publication timelines. Please note that, though access restrictions are acceptable now, your entire data will need to be made freely accessible if your manuscript is accepted for publication. This policy applies to all data except where public deposition would breach compliance with the protocol approved by your research ethics board. If you are unable to adhere to our open data policy, please kindly revise your statement to explain your reasoning and we will seek the editor's input on an exemption. Please be assured that, once you have provided your new statement, the assessment of your exemption will not hold up the peer review process.

3. We note you have included a table to which you do not refer in the text of your manuscript. Please ensure that you refer to Table 1 in your text; if accepted, production will need this reference to link the reader to the Table.

4. If the reviewer comments include a recommendation to cite specific previously published works, please review and evaluate these publications to determine whether they are relevant and should be cited. There is no requirement to cite these works unless the editor has indicated otherwise.

5. Please review your reference list to ensure that it is complete and correct. If you have cited papers that have been retracted, please include the rationale for doing so in the manuscript text, or remove these references and replace them with relevant current references. Any changes to the reference list should be mentioned in the rebuttal letter that accompanies your revised manuscript. If you need to cite a retracted article, indicate the article’s retracted status in the References list and also include a citation and full reference for the retraction notice.

[Note: HTML markup is below. Please do not edit.]

Reviewers' comments:

Reviewer's Responses to Questions

**Comments to the Author**

1. Does the manuscript provide a valid rationale for the proposed study, with clearly identified and justified research questions?

Reviewer #1: Yes

Reviewer #2: Yes

Reviewer #3: Yes

**********

2. Is the protocol technically sound and planned in a manner that will lead to a meaningful outcome and allow testing the stated hypotheses?

Reviewer #1: Yes

Reviewer #2: Yes

Reviewer #3: Yes

**********

3. Is the methodology feasible and described in sufficient detail to allow the work to be replicable?

Reviewer #1: Yes

Reviewer #2: Yes

Reviewer #3: Yes

**********

4. Have the authors described where all data underlying the findings will be made available when the study is complete?

The PLOS Data policy

Reviewer #1: Yes

Reviewer #2: Yes

Reviewer #3: Yes

**********

5. Is the manuscript presented in an intelligible fashion and written in standard English?

Reviewer #1: Yes

Reviewer #2: Yes

Reviewer #3: Yes

**********

Please use the space provided to explain your answers to the questions above and, if applicable, provide comments about issues authors must address before this protocol can be accepted for publication. You may also include additional comments for the author, including concerns about research or publication ethics.

You may also provide optional suggestions and comments to authors that they might find helpful in planning their study.

Reviewer #1: Well written protocol

Well written protocolWell written protocolWell written protocolWell written protocolWell written protocolWell written protocolWell written protocolWell written protocolWell written protocolWell written protocolWell written protocolWell written protocolWell written protocolWell written protocolWell written protocolWell written protocolWell written protocolWell written protocolWell written protocolWell written protocolWell written protocolWell written protocolWell written protocolWell written protocolWell written protocolWell written protocolWell written protocolWell written protocolWell written protocolWell written protocol9

Reviewer #2: This is a well-designed systematic review adhering to the PRISMA-P protocol and registered with PROSPERO. It addresses an important clinical question.

I note the authors have made reference to the limits that may be possible with meta-analysis. My main concern is that there may be a number of trials comparing differing airway techniques, which fails to provide a baseline control for comparative studies (eg studies comparing a videolaryngoscope to direct laryngoscopy versus studies comparing different laryngoscopes) and comparison across techniques may not be straightforward. It is also common in airway studies for different primary and secondary outcomes to be used, again which the authors refer to, but which may be methodologically limiting.

It may be also worth consideration that many measured outcomes may not be fully useful in describing airway management effectiveness (for example, if one intervention has a mean time to intubation of 65 seconds but a 90% success rate compared to neither with a mean time of 50 seconds, but only a 65% success rate, comparison is complex) and some discussion of how this will be accounted for would be worthwhile.

Reviewer #3: Dear Authors,

Your basic question is appropriate to improve the difficulties in the anaesthetic management of children with cleft lip and/or palate. The methods seem to be correct for this job.

**********

what does this mean?). If published, this will include your full peer review and any attached files.

If you choose “no”, your identity will remain anonymous but your review may still be made public.

**Do you want your identity to be public for this peer review?**  For information about this choice, including consent withdrawal, please see our Privacy Policy

Reviewer #1: No

Reviewer #2: No

Reviewer #3: **Yes:**  Istvan Batai

**********

[NOTE: If reviewer comments were submitted as an attachment file, they will be attached to this email and accessible via the submission site. Please log into your account, locate the manuscript record, and check for the action link "View Attachments". If this link does not appear, there are no attachment files.]

To ensure your figures meet our technical requirements, please review our figure guidelines: https://journals.plos.org/plosone/s/figures

You may also use PLOS’s free figure tool, NAAS, to help you prepare publication quality figures: https://journals.plos.org/plosone/s/figures#loc-tools-for-figure-preparation.

NAAS will assess whether your figures meet our technical requirements by comparing each figure against our figure specifications.

# Author comment: Author response to Decision Letter 1

**DOI:** 10.1371/journal.pone.0361327.r002

29 Sep 2026

Response to Reviewers

Manuscript ID: PONE-D-26-43191

Title: Airway management and anaesthetic interventions for children with cleft lip and/or palate undergoing general anaesthesia: protocol for a systematic review and meta-analysis

Dear Dr. Silvia Fiorelli and Reviewers,

Thank you for the careful evaluation of our manuscript and for the constructive comments. We have revised the protocol in response to all points raised. Below is our point-by-point response. All changes are highlighted in the “Revised Manuscript with Track Changes” file. A clean version of the revised manuscript is also provided.

Journal Requirements

1. PLOS ONE style requirements

We have carefully reviewed the PLOS ONE formatting templates and revised the manuscript (title page, headings, spacing, and overall formatting) to ensure full compliance with the journal’s style requirements. In addition, the name of the reporting guideline for protocols has been corrected to “(PRISMA-P) 2015” (Methods: Study design and reporting framework).

2. Data Availability Statement

We have clarified our data-sharing plan. Upon completion of the systematic review, the study-level extraction dataset, risk-of-bias assessments, and data underlying any meta-analytic estimates will be made available as Supporting Information. The Data Availability Statement in the manuscript has been updated accordingly.

3. Reference to Table 1

We thank the editors for pointing this out. We have now explicitly referred to Table 1 in the main text (Search strategy section). The sentence now reads:

The detailed search terms and Medical Subject Heading (MeSH) strategies used are presented in Table 1.

4. Recommended citations

None of the reviewers recommended citation of specific additional works. No changes were required.

5. Reference list

We have carefully reviewed the reference list for completeness, accuracy, and currency. No retracted articles were identified. No changes to the reference list were required.

Reviewer #1

We thank Reviewer #1 for the positive assessment of the protocol.

Reviewer #2

We sincerely thank Reviewer #2 for the thoughtful and constructive comments regarding the methodological challenges of synthesising heterogeneous airway trials.

Comment:

The reviewer noted that many trials compare different airway techniques without a consistent baseline control, that primary and secondary outcomes often differ across studies, and that outcomes such as time to intubation and success rate can be complex to interpret together. The reviewer suggested discussion of how these issues will be handled.

Response:

We fully agree with these important points. In response, we have substantially strengthened the Data synthesis section to make our planned approach explicit:

• Studies will be grouped for quantitative synthesis only when interventions and comparators are judged sufficiently similar.

• Trials comparing different classes of devices without a shared control arm will not be pooled in a single pairwise meta-analysis.

• Where data allow, exploratory subgroup analyses by intervention category (e.g., videolaryngoscopy versus direct laryngoscopy, specific device designs, or anaesthetic strategies) will be considered.

• When quantitative synthesis is inappropriate because of clinical diversity, results will be presented narratively and in structured tables organised by intervention type and outcome.

• Continuous outcomes such as time to intubation will be interpreted jointly with dichotomous success-related outcomes (first-attempt success, number of attempts, and failed intubation). A shorter mean intubation time will not be interpreted in isolation if accompanied by lower success rates or higher complication rates.

• Network meta-analysis will not be undertaken because the evidence network is expected to be sparse, and trials without a shared comparator will not be combined.

• Time to intubation, success-related outcomes and airway complications will be tabulated side by side for each comparison, so that speed and success can be judged together.

• A corresponding sentence has been added to the limitations paragraph of the Discussion.

These additions directly address the reviewer’s concerns about heterogeneity of techniques, lack of a common baseline, and the clinical interpretation of time versus success outcomes. We believe the revised synthesis plan is now more transparent and methodologically robust.

Reviewer #3

We thank Reviewer #3 for the positive evaluation of the research question and methods.

We hope that the revisions satisfactorily address all comments. We remain grateful for the reviewers’ and editor’s time and expertise, which have improved the clarity and methodological rigour of this protocol.

Kind regards,

Kazi Mahzabin Arin

(on behalf of all co-authors)

Corresponding author

Department of Anaesthesia, Analgesia and Intensive Care Medicine

Bangladesh Medical University, Shahbag, Dhaka, Bangladesh

Email: kazimahzabinarin@bsmmu.edu.bd

Attachment Submitted filename: Response to Reviwers.docx

# Peer review: Decision Letter 1

**DOI:** 10.1371/journal.pone.0361327.r003

**Authors:** Silvia Fiorelli

8 Oct 2026

Airway management and anaesthetic interventions for children with cleft lip and/or palate undergoing general anaesthesia: protocol for a systematic review and meta-analysis

PONE-D-26-43191R1

Dear Dr. Kazi Mahzabin Arin,

We’re pleased to inform you that your manuscript has been judged scientifically suitable for publication and will be formally accepted for publication once it meets all outstanding technical requirements.

Within one week, you’ll receive an e-mail detailing the required amendments. When these have been addressed, you’ll receive a formal acceptance letter and your manuscript will be scheduled for publication.

An invoice will be generated when your article is formally accepted. Please note, if your institution has a publishing partnership with PLOS and your article meets the relevant criteria, all or part of your publication costs will be covered. Please make sure your user information is up-to-date by logging into Editorial Manager at Editorial Manager® and clicking the ‘Update My Information' link at the top of the page. For questions related to billing, please contact billing support.

If your institution or institutions have a press office, please notify them about your upcoming paper to help maximize its impact. If they’ll be preparing press materials, please inform our press team as soon as possible -- no later than 48 hours after receiving the formal acceptance. Your manuscript will remain under strict press embargo until 2 pm Eastern Time on the date of publication. For more information, please contact onepress@plos.org.

Kind regards,

Silvia Fiorelli

Academic Editor

PLOS One

Additional Editor Comments (optional):

Reviewers' comments:

Reviewer's Responses to Questions

**Comments to the Author**

1. Does the manuscript provide a valid rationale for the proposed study, with clearly identified and justified research questions?

Reviewer #2: Yes

**********

2. Is the protocol technically sound and planned in a manner that will lead to a meaningful outcome and allow testing the stated hypotheses?

Reviewer #2: Yes

**********

3. Is the methodology feasible and described in sufficient detail to allow the work to be replicable?

Reviewer #2: Yes

**********

4. Have the authors described where all data underlying the findings will be made available when the study is complete?

The PLOS Data policy

Reviewer #2: Yes

**********

5. Is the manuscript presented in an intelligible fashion and written in standard English?

Reviewer #2: Yes

**********

Please use the space provided to explain your answers to the questions above and, if applicable, provide comments about issues authors must address before this protocol can be accepted for publication. You may also include additional comments for the author, including concerns about research or publication ethics.

You may also provide optional suggestions and comments to authors that they might find helpful in planning their study.

Reviewer #2: This was already a strong submission, and the additional modifications are more than satisfactory. The researchers should be commended on a robust methodology and addressing a clinically important question. I would strongly support publication.

**********

what does this mean?). If published, this will include your full peer review and any attached files.

If you choose “no”, your identity will remain anonymous but your review may still be made public.

**Do you want your identity to be public for this peer review?**  For information about this choice, including consent withdrawal, please see our Privacy Policy

Reviewer #2: No

**********

# Editor report: Acceptance letter

**DOI:** 10.1371/journal.pone.0361327.r004

**Authors:** Silvia Fiorelli

PONE-D-26-43191R1

PLOS One

Dear Dr. Arin,

I'm pleased to inform you that your manuscript has been deemed suitable for publication in PLOS One. Congratulations! Your manuscript is now being handed over to our production team.

At this stage, our production department will prepare your paper for publication. This includes ensuring the following:

* All references, tables, and figures are properly cited

* All relevant supporting information is included in the manuscript submission,

* There are no issues that prevent the paper from being properly typeset

You will receive further instructions from the production team, including instructions on how to review your proof when it is ready. Please keep in mind that we are working through a large volume of accepted articles, so please give us a few days to review your paper and let you know the next and final steps.

Lastly, if your institution or institutions have a press office, please let them know about your upcoming paper now to help maximize its impact. If they'll be preparing press materials, please inform our press team within the next 48 hours. Your manuscript will remain under strict press embargo until 2 pm Eastern Time on the date of publication. For more information, please contact onepress@plos.org.

You will receive an invoice from PLOS for your publication fee after your manuscript has reached the completed accept phase. If you receive an email requesting payment before acceptance or for any other service, this may be a phishing scheme. Learn how to identify phishing emails and protect your accounts at https://explore.plos.org/phishing.

If we can help with anything else, please email us at customercare@plos.org.

Thank you for submitting your work to PLOS One and supporting open access.

Kind regards,

PLOS One Editorial Office Staff

on behalf of

Dr. Silvia Fiorelli

Academic Editor

PLOS One
